# Cyclic di-AMP Acts as an Extracellular Signal That Impacts *Bacillus subtilis* Biofilm Formation and Plant Attachment

**DOI:** 10.1128/mBio.00341-18

**Published:** 2018-03-27

**Authors:** Loni Townsley, Sarah M. Yannarell, Tuanh Ngoc Huynh, Joshua J. Woodward, Elizabeth A. Shank

**Affiliations:** a Department of Biology, University of North Carolina at Chapel Hill, Chapel Hill, North Carolina, USA; b Department of Microbiology and Immunology, University of North Carolina at Chapel Hill, Chapel Hill, North Carolina, USA; c Department of Microbiology, University of Washington, Seattle, Washington, USA; Mass General Hospital

**Keywords:** *Arabidopsis thaliana*, *Bacillus subtilis*, biofilms, cell-cell interaction, cyclic di-AMP, plant-microbe interactions

## Abstract

There is a growing appreciation for the impact that bacteria have on higher organisms. Plant roots often harbor beneficial microbes, such as the Gram-positive rhizobacterium *Bacillus subtilis*, that influence their growth and susceptibility to disease. The ability to form surface-attached microbial communities called biofilms is crucial for the ability of *B. subtilis* to adhere to and protect plant roots. In this study, strains harboring deletions of the *B. subtilis* genes known to synthesize and degrade the second messenger cyclic di-adenylate monophosphate (c-di-AMP) were examined for their involvement in biofilm formation and plant attachment. We found that intracellular production of c-di-AMP impacts colony biofilm architecture, biofilm gene expression, and plant attachment in *B. subtilis*. We also show that *B. subtilis* secretes c-di-AMP and that putative c-di-AMP transporters impact biofilm formation and plant root colonization. Taken together, our data describe a new role for c-di-AMP as a chemical signal that affects important cellular processes in the environmentally and agriculturally important soil bacterium *B. subtilis*. These results suggest that the “intracellular” signaling molecule c-di-AMP may also play a previously unappreciated role in interbacterial cell-cell communication within plant microbiomes.

## IMPORTANCE

Plants harbor bacterial communities on their roots that can significantly impact their growth and pathogen resistance. In most cases, however, the signals that mediate host-microbe and microbe-microbe interactions within these communities are unknown. A detailed understanding of these interaction mechanisms could facilitate the manipulation of these communities for agricultural or environmental purposes. *Bacillus subtilis* is a plant-growth-promoting bacterium that adheres to roots by forming biofilms. We therefore began by exploring signals that might impact its biofilm formation. We found that *B. subtilis* secretes c-di-AMP and that the ability to produce, degrade, or transport cyclic di-adenylate monophosphate (c-di-AMP; a common bacterial second messenger) affects *B. subtilis* biofilm gene expression and plant attachment. To our knowledge, this is the first demonstration of c-di-AMP impacting a mutualist host-microbe association and suggests that c-di-AMP may function as a previously unappreciated extracellular signal able to mediate interactions within plant microbiomes.

## INTRODUCTION

Plant roots and leaves harbor rich microbial ecosystems comprised of bacteria and fungi that are crucial for plant health (1). *Bacillus subtilis* is a Gram-positive rhizobacterium that has been shown to colonize a multitude of plant species (2–4). The exudates from *Arabidopsis thaliana* roots selectively signal to and recruit *B. subtilis* cells (5, 6), which utilize chemotaxis machinery and flagellar motility to move by chemotaxis to the root surface (7). Biologically active compounds secreted by *B. subtilis* promote plant growth and elicit induced systemic resistance (ISR) in plants (8, 9); this bacterium is often used as a biocontrol agent to protect plants from disease (10, 11). In addition, plant root colonization is beneficial to the bacteria because root exudates provide a rich fixed-carbon source (12). These interkingdom interactions are highly relevant to environmental ecology and agriculture.

Biofilm formation is essential for the attachment of *B. subtilis* to plant roots (2) and for conferring protection against plant pathogens (13). Biofilms are aggregates of cells or surface-attached microbial communities encased in a self-produced extracellular matrix. Plant-produced compounds such as plant polysaccharides can induce biofilm formation at the root surface (2), and the plant pheromone methyl salicylate can impact biofilm architecture in *B. subtilis* (14). Within biofilms, *B. subtilis* differentiates into multiple cell types, including matrix-producing, surfactin-producing, sporulating, and motile cells, which localize to distinct regions of the community (15). The main structural components of the *B. subtilis* biofilm matrix are an exopolysaccharide (EPS) and two proteins: TasA, an amyloid-like protein that forms long extracellular filaments that provide structural integrity to the biofilm (16); and BslA, a biofilm surface layer protein that confers hydrophobicity to the structure (17). These matrix components are encoded by the *epsA*-*epsO* (*epsA-O*) operon, the *tapA* operon, and the *bslA* gene, respectively.

Bacteria commonly use cyclic dinucleotides to relay environmental signals to downstream receptors that modulate a variety of cellular processes important for survival. Cyclic di-guanylate monophosphate (c-di-GMP) is a broadly conserved dinucleotide produced by bacteria and archaea (18, 19) that is involved in processes such as fatty acid synthesis, growth under low-potassium conditions, DNA integrity sensing, and cell wall homeostasis (20). C-di-AMP is synthesized by diadenylate cyclases (DACs) and is degraded by phosphodiesterases (PDEs). *Bacillus subtilis* has three DACs (CdaA, CdaS, and DisA), which contain conserved DAC domains (19, 21), and two PDEs (GdpP and PgpH), which contain catalytic DHH/DHHA1 (Asp-His-His) and HD (His-Asp) domains, respectively (22, 23). C-di-AMP is an essential second messenger in *B. subtilis*, and yet accumulation to high levels can be lethal and can lead to the emergence of suppressor mutations (24–26), indicating that c-di-AMP homeostasis is finely tuned within *B. subtilis* cells. Previous studies have demonstrated that the bacterial pathogens *Listeria monocytogenes* (27), *Mycobacterium tuberculosis* (28), and *Chlamydia trachomatis* (29) secrete c-di-AMP into liquid media as well as host cytosol, where it induces a robust type I interferon (IFN) response (27–29). The role of c-di-AMP secretion in this process has not been completely elucidated (26). It also remains unknown whether bacteria can sense or respond to extracellular c-di-AMP.

In this study, we demonstrated that c-di-AMP signaling plays an important role in biofilm formation and plant attachment in *B. subtilis* through the phenotypic characterization of *B. subtilis* DAC and PDE mutants. We found that *B. subtilis* secretes c-di-AMP and that c-di-AMP secretion requires two genes (*ycnB* and *yhcA*) that encode predicted permeases that impact biofilm architecture and plant colonization. We show that a *B. subtilis* strain lacking both of these transporters secretes less c-di-AMP and that this defect has a striking impact on plant attachment phenotypes. Thus, our data suggest, to our knowledge for the first time, that extracellular c-di-AMP can be sensed by *B. subtilis* and can affect important cellular processes such as biofilm attachment to plant roots.

## RESULTS

### Mutations that disrupt c-di-AMP synthesis and degradation affect biofilm architecture.

To determine if c-di-AMP signaling impacts biofilm formation, we generated *B. subtilis* NCIB3610 strains lacking the individual genes that encode DACs (*cdaA*, *cdaS*, and *disA*) and PDEs (*gdpP* and *pgpH*). We then performed c-di-AMP measurements in each strain to determine whether c-di-AMP levels were affected as predicted (i.e., whether c-di-AMP levels were lower in the DAC mutant strains and higher in the PDE mutant strains) (see Fig. S1 in the supplemental material).

10.1128/mBio.00341-18.1 FIG S1  Intracellular c-di-AMP levels in *B. subtilis*. C-di-AMP was extracted from whole cells and quantified using HPLC-MS/MS. Error bars represent standard deviations of results from three biological replications. *, *P* < 0.05. Download FIG S1, EPS file, 0.7 MB. Copyright © 2018 Townsley et al. 2018 Townsley et al. This content is distributed under the terms of the Creative Commons Attribution 4.0 International license.

**FIG 1:**
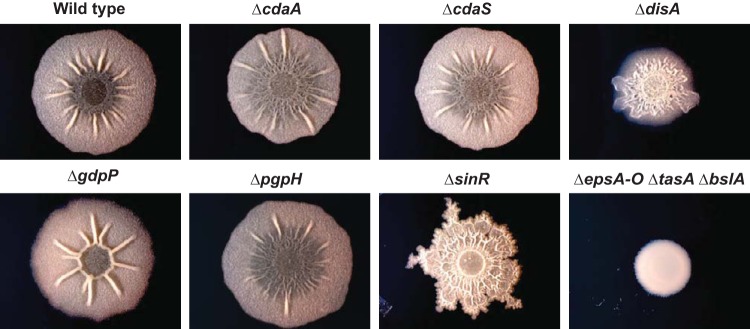
Colony morphology of *B. subtilis* harboring deletions of genes encoding DACs and PDEs. Representative images show biofilm architecture for *B. subtilis* NCIB 3610, DAC mutants (Δ*cdaA*, Δ*cdaS*, and Δ*disA*), PDE mutants (Δ*gdpP* and Δ*pgpH*), and known biofilm mutants (Δ*sinR* and Δ*epsA-O* Δ*tasA* Δ*bslA*) grown on the biofilm-inducing medium MSgg for 48 h.

In *B. subtilis*, colony morphology is impacted by biofilm matrix production. Thus, to determine if biofilm formation was impacted in these mutants, the colony morphology of each strain was evaluated after 48 h of growth at 30°C on MSgg medium (5 mM potassium phosphate [pH 7], 100 mM morpholinepropanesulfonic acid [MOPS; pH 7], 2 mM MgCl_2_, 700 μM CaCl_2_, 50 μM MnCl_2_, 50 μM FeCl_3_, 1 μM ZnCl_2_, 2 μM thiamine, 0.5% glycerol, 0.5% glutamate) (a biofilm-inducing medium) agar plates. For comparison, a strain lacking the biofilm repressor *sinR* and a strain lacking all the biofilm matrix genes (*epsA-O*, *tasA*, and *bslA*) were used as controls for high- and low-biofilm-matrix producers, respectively. Δ*cdaA* and Δ*cdaS* exhibited small but reproducible differences in colony morphology compared with wild-type *B. subtilis* (Fig. 1), whereas the Δ*disA* mutant exhibited a strikingly altered colony morphology on MSgg medium (Fig. 1). The PDE mutant Δ*gdpP* displayed a star-shaped colony morphology with large wrinkles connecting in a raised circle pattern at the center, while the PDE mutant Δ*pgpH* produced colonies with a flatter profile and wrinkles that were less pronounced than those seen with the wild type (Fig. 1). Since the Δ*disA*, Δ*gdpP*, and Δ*pgpH* strains exhibited the most dramatic biofilm phenotypes, we focused on these mutants in further characterizing the role that c-di-AMP plays in biofilm formation in *B. subtilis*.

### Biofilm gene expression.

**FIG 2:**
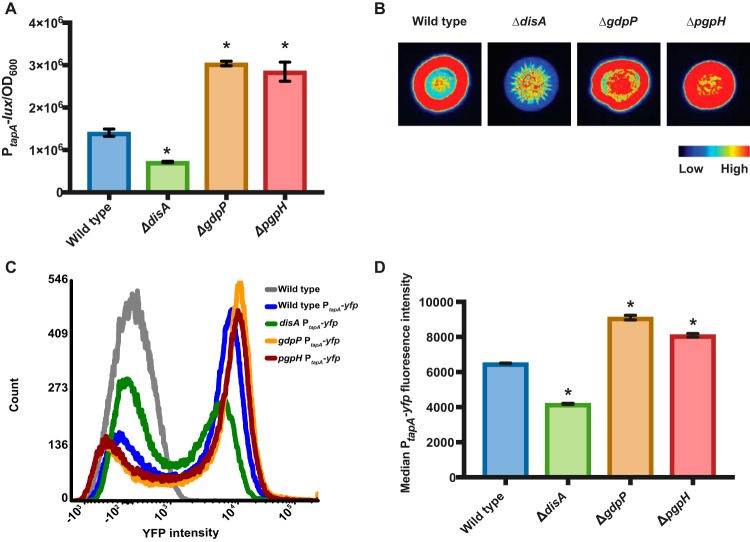
Biofilm gene expression in *B. subtilis* DAC (Δ*disA*) and PDE (Δ*gdpP* and Δ*pgpH*) mutants. (A) *B. subtilis* Δ*disA*, Δ*gdpP*, and Δ*pgpH* mutants were grown in MSgg liquid cultures, and the promoter activity of *tapA* was monitored by luminescence produced from the P*_tapA_-luxABCDE* construct in each of these strains after 24 h. (B) The promoter activity of *tapA* in colony biofilms was similarly monitored after 24 h of growth on MSgg agar plates. (C) Flow cytometry of the fluorescence intensity of *B. subtilis* cells harvested from colonies grown on MSgg at 24 h. A total of 50,000 cells were quantified for each sample. (D) Median P*_tapA_-yfp* fluorescence intensities of *B. subtilis* Δ*disA*, Δ*gdpP*, and Δ*pgpH* cells harvested from colonies grown on MSgg at 24 h. Error bars indicate standard deviations of results from three biological replications. *, *P* < 0.05.

To determine if *disA*, *gdpP*, and *pgpH* impact biofilm formation through modulation of biofilm matrix gene expression, we deleted each gene of interest in a *B. subtilis* strain containing a luciferase reporter for biofilm gene expression. This strain harbored the *luxABCDE* operon driven by the *tapA* promoter (P*_tapA_-lux*) integrated into the neutral *sacA* locus in the chromosome (30). Luminescence measurements were taken from shaking cultures of these strains grown in MSgg liquid media at 24 h. Under the conditions examined, *tapA* promoter activity in the Δ*disA* mutant was lower than that seen with the wild type and *tapA* promoter activity in the Δ*gdpP* and Δ*pgpH* mutants was higher than that seen with the wild type (Fig. 2A). These results indicate that biofilm matrix gene expression was decreased in mutant Δ*disA* relative to wild-type *B. subtilis* and was generally increased in mutants Δ*gdpP* and Δ*pgpH*, consistent with c-di-AMP levels impacting the expression of biofilm matrix genes. Shaken liquid cultures are ideal for quantitative luminescence measurements; however, gene expression levels often differ between planktonic and biofilm-grown cells. To observe P_*tapA*_-*lux* in colony biofilms, these strains were spotted onto MSgg agar plates and P*_tapA_-lux* was detected after growth using chemiluminescent imaging. We found that the promoter activity was highest at the edges of the colonies in the wild type (Fig. 2B). Consistent with the liquid culture data, at the colony level, *tapA* promoter activity appeared to be lower overall in the Δ*disA* mutant than in the wild type (Fig. 2B) and was higher overall in the Δ*gdpP* and Δ*pgpH* mutants than in the wild type (Fig. 2B).

Since *B. subtilis* P_*tapA*_ expression localized to different areas of the biofilm in wild-type and mutant *B. subtilis* colonies, we wanted to quantify the percentage of matrix-producing cells within each population. We used flow cytometry to quantify fluorescent cells in wild-type, Δ*disA*, Δ*pgpH*, and Δ*gdpP* colonies containing the P*_tapA_-yfp* reporter (*yfp* encodes yellow fluorescent protein [YFP]). We harvested biofilm colonies grown on MSgg medium for 24 h and fixed cells with paraformaldehyde. To quantify fluorescent cells, we performed gating on a sample of *B. subtilis* cells constitutively expressing YFP. These data show that the percentage of Δ*disA* cells expressing P_*tapA*_-*yfp* (46%) was lower than the percentage of wild-type *B. subtilis* cells (69%) (Fig. 2C). The percentage of cells expressing the P_*tapA*_-*yfp* biofilm reporter within the Δ*pgpH* and Δ*gdpP* biofilm colonies was similar to that seen with wild-type *B. subtilis* (68% and 73%, respectively) (Fig. 2C). Notably, however, a greater median fluorescence intensity was observed in the Δ*pgpH* and Δ*gdpP* strains than in the wild-type strain. These data indicate that although similar percentages of cells were fluorescent in these strains, the fluorescent cells in the PDE mutants were expressing higher levels of *yfp* (i.e., were expressing P_*tapA*_ more strongly) than the fluorescent wild-type cells (Fig. 2D). Taken together, these results imply that *disA*, *gdpP*, and *pgpH* are all involved in modulating biofilm formation by altering *tapA* biofilm gene expression.

### Complementation of *disA*, *gdpP*, and *pgpH*.

We then wanted to confirm that the observed changes in *tapA* promoter activity in the Δ*disA*, Δ*gdpP*, and Δ*pgpH* strains were directly attributable to the disruption of these genes. To do so, we complemented each of these mutant strains with a single copy of an IPTG (isopropyl-β-d-thiogalactopyranoside)-inducible copy of their cognate wild-type gene in the *amyE* site of the chromosome (31), with the expectation that (if these genes were responsible for the effects on *tapA* promoter activity) the complemented strains would exhibit P_*tapA*_-*lux* activity more similar to wild-type levels than the uncomplemented strains. Each of these strains also harbored P_*tapA*_-*lux*. The *disA* complementation strain showed a small but reproducible increase in P_*tapA*_-*lux* activity relative to the levels observed in the Δ*disA* mutant, while the PDE complementation strains showed decreases in P_*tapA*_-*lux* activity relative to the corresponding deletion strain (Fig. S2). These results confirm the respective roles of these genes in c-di-AMP-mediated biofilm formation.

10.1128/mBio.00341-18.2 FIG S2  P*_tapA_-lux* expression in complementation strains. P*_tapA_-lux* levels were determined in the wild-type, Δ*disA*, Δ*gdpP*, and Δ*pgpH* strains, as well as in each of the mutant strains also harboring a single copy of an IPTG-inducible copy of the cognate wild-type gene inserted into the *amyE* site of the chromosome grown at 28°C for 24 h in MSgg with the addition of 1 mM IPTG. Error bars represent standard deviations of results of two biological replications. *, *P* < 0.05. Download FIG S2, EPS file, 0.9 MB. Copyright © 2018 Townsley et al. 2018 Townsley et al. This content is distributed under the terms of the Creative Commons Attribution 4.0 International license.

### Surfactin production.

**FIG 3:**
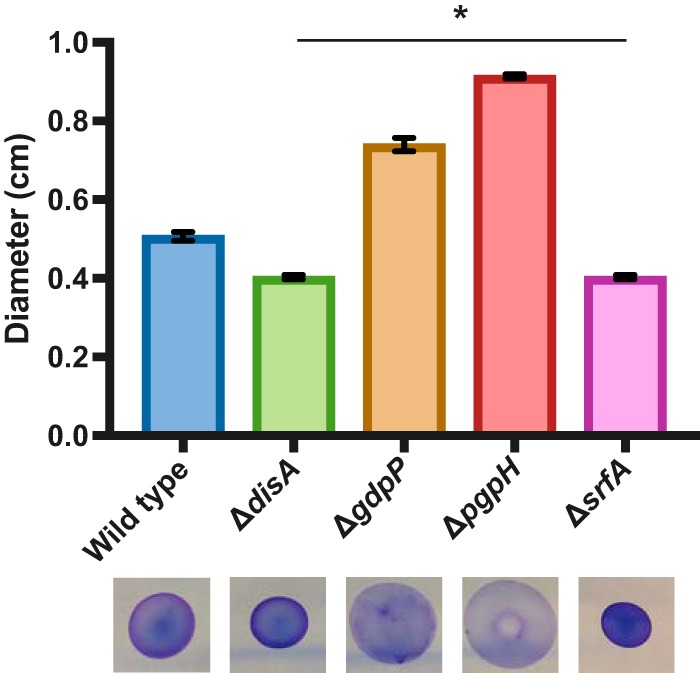
Surfactin production in DAC (Δ*disA*) and PDE (Δ*gdpP* and Δ*pgpH*) mutants. Surfactin production was detected by a drop-collapse assay that measured the diameter of a drop of spent media containing 0.1% crystal violet dye for detection. The average diameters of the drops of spent media from three biological replications are quantified in the bar graph; representative images of the collapsed drops are shown at the bottom. *, *P* < 0.05.

Previous studies have demonstrated that, in addition to matrix gene expression, surfactin production is relevant to biofilm architecture in *B. subtilis* (32, 33). To determine if surfactin production was altered in the DAC and PDE mutants, we performed a drop-collapse assay using cell-free spent media obtained after growing each mutant and wild-type *B. subtilis* in liquid culture overnight. If surfactin is present in the spent medium, it reduces the surface tension of the liquid, allowing it to spread further when spotted onto a hard surface; adding a dye allows the spread of the spent medium to be visualized and measured. A strain harboring a deletion of *srfA*, the locus responsible for surfactin production, was used as a negative control. The Δ*disA* mutant produced less surfactin than the wild type, similar to the Δ*srfA* control, while mutants Δ*gdpP* and Δ*pgpH* both produced more surfactin than the wild type (Fig. 3). Surfactin production in these mutants therefore correlates with the observed biofilm phenotypes and *tapA* promoter activity.

### C-di-AMP production affects plant attachment.

**FIG 4:**
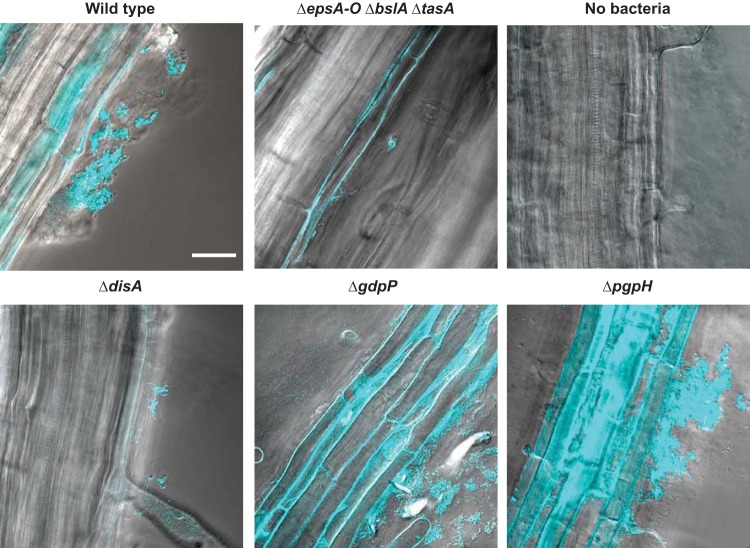
Plant root attachment is affected by mutations altering c-di-AMP production and degradation. Wild-type and c-di-AMP mutant strains constitutively expressing mTurquoise were incubated with 6-day-old *A. thaliana* seedlings for 24 h. Images of bacterial associations with the plant roots were obtained by confocal fluorescence microscopy. Panels show overlays of differential interference contrast and fluorescent images where the fluorescent cells are falsely colored blue. Bar, 50 µm.

Biofilm formation is crucial for *B. subtilis* attachment to plant roots (2). We therefore hypothesized that since these c-di-AMP mutants exhibited altered biofilm phenotypes, they might also impact plant attachment. To test this prediction, we examined whether the c-di-AMP mutants exhibited altered attachment to *Arabidopsis thaliana* roots. Six-day-old *A. thaliana* seedlings were added to media containing *B. subtilis* strains constitutively producing the fluorescent protein mTurquoise in 48-well plates, and bacterial attachment to the roots was imaged using confocal laser scanning microscopy after 24 h. In addition to the wild-type strain and the Δ*disA*, Δ*gdpP*, and Δ*pgpH* mutants, we examined a biofilm matrix deletion mutant known to be unable to colonize plant roots (mutant Δ*epsA-O* Δ*tasA* Δ*bslA*) (2). The Δ*disA* mutant displayed a severe colonization defect, similar to the results seen with the matrix-deletion control (Fig. 4), while the strains lacking either PDE gene (mutants Δ*gdpP* and Δ*pgpH*) both colonized better than the wild type (Fig. 4). We observed the same trends when bacteria were recovered from the roots and CFU were counted (Fig. S3). These results are consistent with the respective biofilm phenotypes observed as described above and indicate that c-di-AMP signaling is important for *B. subtilis* plant attachment.

10.1128/mBio.00341-18.3 FIG S3  Plant root attachment by c-di-AMP mutants. Wild-type and c-di-AMP mutant strains were incubated with 6-day-old *A. thaliana* seedlings for 24 h, and then roots were gently washed and subjected to vigorous vortex mixing in fresh medium, which was used to plate serial dilutions. CFUs were counted from the plant root. Error bars represent standard deviations of results from three biological replications. *, *P* < 0.05; n.s., not significant (*P* > 0.05). Download FIG S3, PDF file, 0.3 MB. Copyright © 2018 Townsley et al. 2018 Townsley et al. This content is distributed under the terms of the Creative Commons Attribution 4.0 International license.

### C-di-AMP secretion contributes to *B. subtilis* biofilm formation.

**FIG 5:**
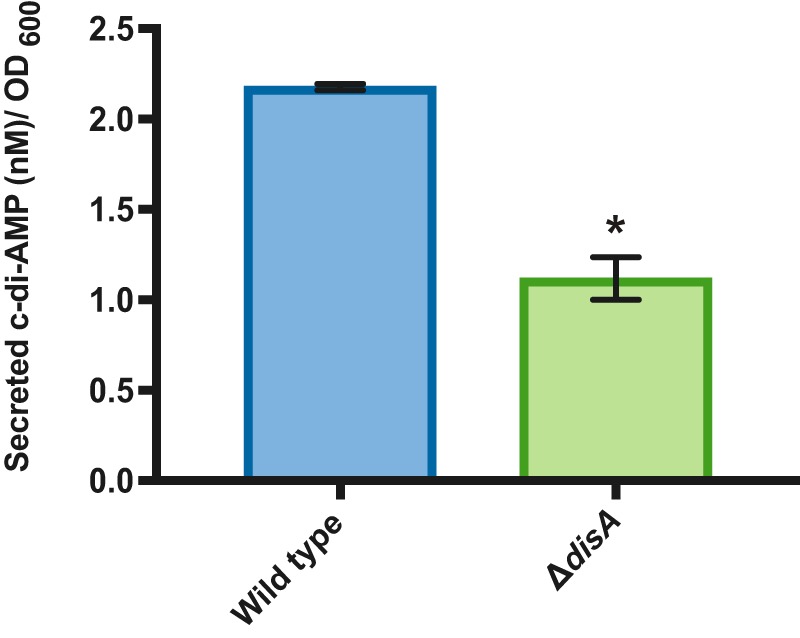
*B. subtilis* secretes c-di-AMP. Secreted c-di-AMP was quantified in the wild-type and Δ*disA* strains using HPLC-MS/MS. Error bars represent standard deviations of results from three biological replications. *, *P* < 0.05.

C-di-AMP has been previously demonstrated to be secreted in a variety of bacterial pathogens (27–29). To address whether *B. subtilis* can secrete c-di-AMP, we directly quantified extracellular concentrations of c-di-AMP using liquid chromatography-mass spectrometry (LC-MS). First, we confirmed that the Δ*disA* mutant did not have a growth defect (Fig. S4). We then detected c-di-AMP in the supernatant of wild-type *B. subtilis* (Fig. 5), and, to a lesser extent, in that of the Δ*disA* mutant grown in liquid culture, indicating that *B. subtilis* indeed secretes c-di-AMP.

10.1128/mBio.00341-18.4 FIG S4  No growth defect was observed in the Δ*disA* mutant. A growth curve comparing wild-type and Δ*disA* strain results over time is shown. Error bars represent standard deviations of results from three biological replications. Download FIG S4, EPS file, 0.9 MB. Copyright © 2018 Townsley et al. 2018 Townsley et al. This content is distributed under the terms of the Creative Commons Attribution 4.0 International license.

**FIG 6:**
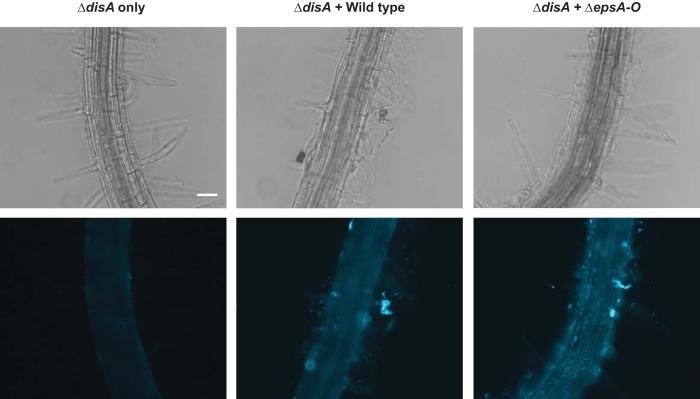
Plant attachment in mutant Δ*disA* is complemented by the addition of wild-type and Δ*epsA-O* strains. The Δ*disA* mutant constitutively expressing mTurquoise was incubated with 6-day-old *A. thaliana* seedlings. Phase-contrast (top) and fluorescence (bottom) images of (A) attachment of the Δ*disA* mutant incubated alone and Δ*disA* mutant attachment under conditions of coincubation with either (B) wild-type *B. subtilis* or (C) the non-matrix-producing Δ*epsA-O* mutant are shown. Bar, 50 µm.

We then hypothesized that, if extracellular secretion and sensing of c-di-AMP were important for *B. subtilis* biofilm formation, the plant attachment defect of Δ*disA* could be a result of its lower c-di-AMP secretion. To determine whether low extracellular levels of c-di-AMP were contributing to the inability of the Δ*disA* mutant to colonize plant roots, we tested whether its attachment defect could be complemented by wild-type *B. subtilis*, which secretes higher levels of c-di-AMP. We performed coculture root inoculations with the Δ*disA* mutant (constitutively expressing mTurquoise) with nonfluorescent wild-type cells; we mixed the cells 1:1 and inoculated plant roots as described above. Root attachment was imaged 24 h after plant inoculation. We found that the Δ*disA* mutant was able to attach to plant roots when wild-type *B. subtilis* was present (Fig. 6). This suggests that the mutant Δ*disA* plant colonization defect can be complemented by the presence of wild-type *B. subtilis* cells.

One trivial explanation for this effect of wild-type *B. subtilis* cells on the ability of mutant Δ*disA* to attach to plant roots could be that cells of the biofilm-deficient Δ*disA* mutant cells simply “stick” to the extracellular matrix that wild-type cells produce. To test this, we cocultured mutant Δ*disA* with the non-matrix-producing Δ*epsA-O* strain and again examined its ability to colonize plant roots. As shown in Fig. 6, the presence of mutant Δ*epsA-O* also allowed mutant Δ*disA* to attach to plant roots, indicating that this complementation is not affected by the ability to produce matrix. Thus, these data suggest that the production of extracellular c-di-AMP by wild-type and Δ*epsA-O* cells may be acting to stimulate biofilm formation in the Δ*disA* cells, allowing them to colonize roots.

### Identification of putative c-di-AMP transporters and their role in biofilm formation.

**FIG 7:**
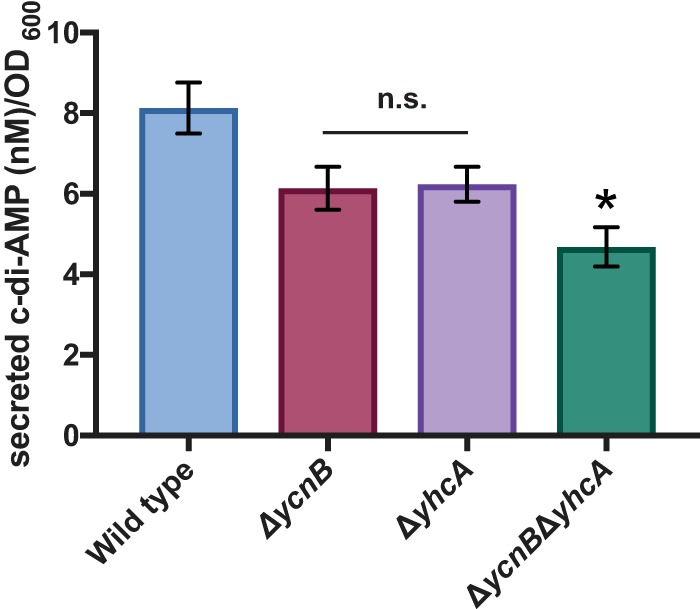
Predicted c-di-AMP transport proteins affect c-di-AMP secretion. Secreted c-di-AMP was quantified in the wild-type, Δ*ycnB*, and Δ*yhcA* strains and in a Δ*ycnB* Δ*yhcA* double mutant. Error bars represent standard deviations of results from at least three biological replications. *, *P* < 0.05; n.s., not significant (*P* > 0.05).

C-di-AMP in *Listeria monocytogenes* is secreted through the multidrug efflux pumps MdrM and MdrT, which are controlled by the regulators MarR and TetR (20). A search of the *B. subtilis* genome for *mdrM* and *mdrT* homologues identified four genes that encode predicted permeases with over 30% identity to both *mdrM* and *mdrT*: *ycnB*, *yhcA*, *imrB* (formerly *yccA*), and *mdtP* (formerly *yusP*) (Table 1). Because *ycnB* and *yhcA* shared the most similarity to the *L. monocytogenes* transporters, we produced strains lacking either *ycnB* or *yhcA* and compared their levels of secreted c-di-AMP to those of the wild type to identify a possible c-di-AMP transporter. We found no significant difference between the wild-type, Δ*ycnB*, and Δ*yhcA* strains in c-di-AMP levels (Fig. 7). Because these putative transporters could potentially compensate for each other, we then produced a double mutant strain lacking both *ycnB* and *yhcA*. We observed a significant decrease in the levels of secreted c-di-AMP in this double mutant strain compared to the wild type (Fig. 7). We did not observe a significant difference in intracellular levels of c-di-AMP in the Δ*ycnB* Δ*yhcA* strain, suggesting that only c-di-AMP secretion (and not c-di-AMP production) is impacted in this strain (Fig. S5).

10.1128/mBio.00341-18.5 FIG S5  *B. subtilis* controls intracellular c-di-AMP levels. Intracellular c-di-AMP was quantified in the wild-type, Δ*ycnB*, and Δ*yhcA* strains and in a Δ*ycnB* Δ*yhcA* double mutant. Error bars represent standard deviations of results from four biological replications. n.s., not significant (*P* > 0.05). Download FIG S5, EPS file, 0.6 MB. Copyright © 2018 Townsley et al. 2018 Townsley et al. This content is distributed under the terms of the Creative Commons Attribution 4.0 International license.

**TABLE 1:** Putative c-di-AMP transporters^a^

Gene no.	Gene name	% protein identity to MdrM	% protein identity to MdrT
BsubsN3_010100002154	*ycnB*	45	54
BsubsN3_010100004934	*yhcA*	42	50
BsubsN3_010100001491	*yccA* or *imrB*	39	43
BsubsN3_010100017762	*yusP* or *mdtP*	31	31

a The *B. subtilis* NCIB 3610 genes listed encode proteins that show sequence similarity to *L. monocytogenes* c-di-AMP transporters MdrM and MdrT.

**FIG 8:**
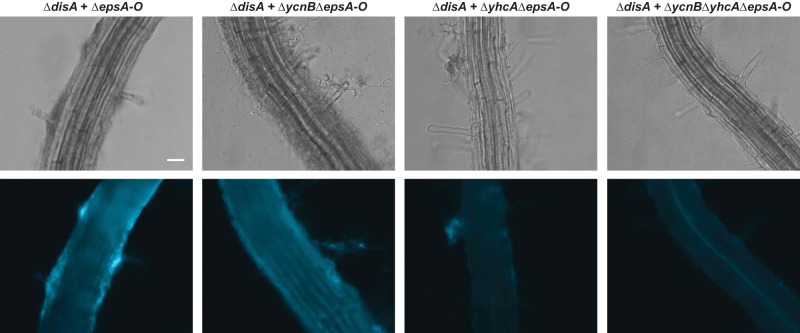
Low c-di-AMP secretion impacts plant root colonization. Six-day-old *A. thaliana* seedlings were incubated with a mutant Δ*disA* strain constitutively expressing mTurquoise for 24 h. Phase-contrast (top) and fluorescence (bottom) representative images are shown of the mutant Δ*disA* attachment seen under conditions of coincubation with non-matrix-producing Δ*epsA-O*, Δ*ycnB* Δ*epsA-O*, Δ*yhcA* Δ*epsA-O*, and Δ*ycnB* Δ*yhcA* Δ*epsA-O* strains from four biological replications. Bar, 25 µm.

We then tested the effects that these putative c-di-AMP transporters had on biofilm formation in the context of plant roots. We cocultured a fluorescent Δ*disA* strain with the transporter mutants on *A. thaliana* roots as described above. Similarly to the data shown in Fig. 6, the Δ*disA* mutant attached to plant roots when it was cocultured with the Δ*epsA-O* mutant (Fig. 8). We then directly tested whether this complementation depended on these transporters by knocking them out of the Δ*epsA-O* strain. The Δ*ycnB* Δ*epsA-O* and Δ*yhcA* Δ*epsA-O* mutants did not complement the attachment defect of Δ*disA* as well as the Δ*epsA-O* mutant alone, and the Δ*disA* mutant had a significant plant colonization defect in the presence of the Δ*ycnB* Δ*yhcA* Δ*epsA-O* mutant (Fig. 8). The extent of Δ*disA* colonization visible in these images is consistent with the quantification of mutant Δ*disA* CFU recovered from the roots (Fig. S6). These results suggest that the *ycnB* and *yhcA* genes are important for the ability of Δ*epsA-O* cells to complement the plant attachment defect of the Δ*disA* mutant and that the double mutant is unable to rescue it. These data are all consistent with a model proposing that the *ycnB* and *yhcA* genes encode c-di-AMP transporters and that their ability to secrete extracellular c-di-AMP impacts biofilm formation and plant attachment in neighboring *B. subtilis* cells.

10.1128/mBio.00341-18.6 FIG S6  Δ*disA* mutant plant root attachment is affected by c-di-AMP transporter mutant strains. Δ*disA* and c-di-AMP transporter mutant strains were coincubated with 6-day-old *A. thaliana* seedlings for 24 h, and then roots were gently washed and sonicated in fresh media, which was used to plate serial dilutions. CFUs were counted from the plant root. Error bars represent standard deviations of results from at least three biological replications. *, *P* < 0.05; n.s., not significant (*P* > 0.05). Download FIG S6, PDF file, 0.3 MB. Copyright © 2018 Townsley et al. 2018 Townsley et al. This content is distributed under the terms of the Creative Commons Attribution 4.0 International license.

## DISCUSSION

Biofilm formation is important for environmental fitness and adaptation in many bacteria. Although diverse mechanisms exist for regulating biofilm formation, cyclic di-nucleotide second messengers play a critical role in many bacteria. The intracellular signaling molecule cyclic di-guanylate monophosphate (c-di-GMP) mediates biofilm formation in a vast number of Gram-negative bacteria (34). C-di-GMP was recently discovered in *B. subtilis* (35, 36); however, unlike its activity in the related Gram-positive bacterium *Bacillus cereus* (37), evidence suggests that c-di-GMP does not play a major role in biofilm formation in *B. subtilis* (35, 36). Emerging studies, however, are indicating that c-di-AMP may be important for controlling biofilm formation in some Gram-positive bacteria; increased levels of intracellular c-di-AMP stimulate biofilm formation in both *Streptococcus mutans* (38) and *Staphylococcus aureus* (39). Here we determined that altering c-di-AMP levels in *B. subtilis*, by deleting either the DACs that synthesize it or the PDEs that degrade it, modulates biofilm formation in *B. subtilis*.

Few previous studies have explored the role of c-di-AMP in *B. subtilis* biofilm formation. One recent study reported that although there was no change in *tapA* and *epsA* expression in single mutants lacking either *gdpP* or *pgpH*, the deletion of both PDEs (which would be predicted to lead to a dramatic accumulation of c-di-AMP) downregulated the mRNA abundance of *tapA* and *epsA* in *B. subtilis* (40). However, transcriptome data from the double PDE mutant in this same study were inconsistent with these results: they showed an upregulation of the biofilm inducer *abh* and a downregulation of the biofilm repressor *abrB*, both of which would be predicted to increase biofilm formation. The study by Gundlach et al. was conducted using growth conditions different from ours, which could have contributed to the discrepancy between the conclusions drawn in our two studies. Our data demonstrate that increased c-di-AMP levels induce the promoter activity of the *tapA* operon that is required for biofilm formation in *B. subtilis*.

Although our data indicate that increases in both intracellular and extracellular levels of c-di-AMP positively influence biofilm formation, we still do not know the molecular details of the mechanisms by which c-di-AMP regulates biofilm formation. One possibility is that c-di-AMP acts through alterations in the phosphorylation state of the master transcriptional regulator Spo0A. A previous study determined that the sporulation delay observed in a *disA* mutant is due to changes in Spo0A phosphorylation (41), although, again, the molecular details of how Spo0A is impacted by c-di-AMP remain unclear. The c-di-AMP receptors identified thus far in *B. subtilis* include two riboswitches that control amino acid transporter gene *ydaO* (renamed *kimA*) (42, 43), the P_II_ signal transducer protein encoded by *darA* (44), and the potassium transport protein KtrA (45). KtrA is part of one of the two main proteins associated with potassium uptake mechanisms in *B. subtilis*: KtrAB and KtrCD (46). When mutated, *ktrC* enhances biofilm formation; potassium leakage is known to induce biofilm formation in *B. subtilis* via the sensor histidine kinase *kinC* (47). Thus, integration of c-di-AMP into the potassium homeostasis network could potentially be a mechanism for impacting biofilm formation in *B. subtilis*. Indeed, the recently renamed YdaO protein (now KimA) has been shown to act as a potassium transporter (42). Interestingly, both *ktrA* and *ktrC* are physically located adjacent to the biofilm-relevant genes in the *B. subtilis* genome: *ktrA* is immediately downstream of *bslA*, while *ktrC* is downstream of *abh* and the *kinC* operon. Additional studies are needed to determine if these or other, yet-to-be-identified receptors are important for connecting c-di-AMP signaling to the biofilm regulatory network in *B. subtilis*.

We also identified two putative c-di-AMP transporters and demonstrated that *B. subtilis* secretes c-di-AMP and can sense and respond to extracellular c-di-AMP. These data suggest an important role for this second messenger in interbacterial communication. To our knowledge, *B. subtilis* is the first nonpathogenic bacterium discovered to secrete c-di-AMP, which implies that this signaling molecule may play a role in bacterial communication not only in human hosts but also in the environment. The biofilm formation and sporulation pathways in *B. subtilis* are controlled by many of the same regulatory elements, and it is believed that sporulation is the culmination of biofilm formation (15). A previous study was able to induce sporulation in *B. subtilis* by the addition of exogenous c-di-AMP (48), further corroborating our observation that *B. subtilis* can sense exogenous c-di-AMP and respond through the biofilm/sporulation regulatory pathway.

Our data are consistent with a model where *B. subtilis* secretion of c-di-AMP impacts biofilm formation and plant attachment in other *B. subtilis* cells. Future studies are needed to test whether *B. subtilis* and other bacteria can sense c-di-AMP produced by other species in the environment and to elucidate the effects that extracellular c-di-AMP production and sensing may have on bacterial community signaling and plant microbiome community structure.

## MATERIALS AND METHODS

### Bacterial strains and growth conditions.

*B. subtilis* NCIB3610 was used as a wild-type strain. *Escherichia coli* DH5α and *B. subtilis* 168 were used for cloning. Overnight cultures were grown on Luria-Bertani (LB)-Lennox medium (10 g of tryptone, 5 g of yeast extract, 5 g of NaCl per liter) at 30°C. Biofilm assays were performed on MSgg medium (5 mM potassium phosphate [pH 7], 100 mM morpholinepropanesulfonic acid [MOPS; pH 7], 2 mM MgCl_2_, 700 μM CaCl_2_, 50 μM MnCl_2_, 50 μM FeCl_3_, 1 μM ZnCl_2_, 2 μM thiamine, 0.5% glycerol, 0.5% glutamate). When needed, chloramphenicol and erythromycin-lincomycin (MLS) were used at 5 μg/ml and 1 μg/ml, respectively.

### Intracellular c-di-AMP quantification.

*B. subtilis* colony biofilms grown on MSgg plates were scraped off, resuspended into 5 ml PBS (phosphate-buffered saline), and sonicated (amplitude = 20 for 12 s with 1-s on/off pulses) to break clumps. Cultures were divided into 4.5 ml (for c-di-AMP quantification) and 500 µl (for protein quantification) portions. The c-di-AMP quantification samples were centrifuged at 4,000 rpm for 20 min and resuspended in 1 ml cold extraction buffer (acetonitrile, methanol, and distilled water [dH_2_O] in a 40:40:20 ratio). Samples were snap-frozen using liquid N_2_ and then incubated at 95°C for 10 min, 0.5 ml of 0.1-mm-diameter glass beads was added to samples, and a FastPrep-24 instrument (MP Biomedicals, Santa Ana, CA, USA) was used to homogenize the samples, treating them at 4 m/s for 45 s twice. Samples were then briefly centrifuged, and the supernatant was recovered and dried using a Savant SC100 SpeedVac (Thermo Fisher Scientific, Waltham, MA). Samples were resuspended in 100 μl liquid LC-MS-grade H_2_O and analyzed using high-performance liquid chromatography–tandem mass spectrometry (HPLC-MS/MS) on a Quantum Ultra triple-quadrupole mass spectrometer (Thermo Fisher Scientific, Waltham, MA) equipped with an Acquity ultraperformance LC (UPLC) separation system (Waters Corp., Milford, MA). An Acquity UPLC HSS T3 column (Waters Corp., Milford, MA) (2.1 mm by 100-mm diameter; 1.8-μm particle size) was used for reverse-phase liquid chromatography. Solvent A was 10 mM ammonium formate–water, and solvent B was 10 mM ammonium formate–methanol. The injection volume was 10 μl, and the flow rate for chromatography was 200 μl/min. A c-di-AMP standard was prepared with purified c-di-AMP (Biolog Life Sciences, Bremen, Germany). C-di-AMP levels were normalized to total protein per milliliter of culture. Protein quantification was performed using the bicinchoninic acid (BCA) assay (Thermo Fisher Scientific, Waltham, MA) with bovine serum albumin (BSA) as standards. Statistical analysis was performed using a one-way analysis of variance (ANOVA) with a Tukey test for multiple comparisons.

### Secreted c-di-AMP quantification.

*B. subtilis* strains were grown in MSgg broth to an optical density at 600 nm (OD_600_) of ~1.0. From these cultures, 0.5-ml samples were collected and centrifuged. The culture supernatants were mixed with heavily labeled (C^13^ N^15^) c-di-AMP in a 1:1 (vol/vol) ratio for mass spectrometry analysis. For extraction of cytoplasmic c-di-AMP from cells grown in liquid culture, cell pellets were resuspended in 50 liters of 0.5 μM heavy-labeled c-di-AMP and then mixed with 500 μl of methanol and sonicated. After centrifugation of the lysed cells, the supernatant was collected as the first fraction. The remaining pellet was resuspended in 50 μl of H_2_O, mixed with 500 μl of methanol, and centrifuged again to collect the supernatant as the second fraction. The two fractions were pooled and evaporated, and the final pellet containing c-di-AMP was resuspended in 50 μl of double-distilled water (ddH_2_O). Mass spectrometry analysis was performed as previously described (23). Statistical analysis was performed using a one-way ANOVA with a Tukey test for multiple comparisons.

### Colony morphology.

*B. subtilis* cells grown overnight on LB-Lennox plates were resuspended in PBS (OD_600_ = 0.5) and then sonicated (amplitude = 20) for 12 s with 1-s on/off pulses. Ten microliters of each culture was then spotted onto MSgg plates and incubated at 30°C for 48 h.

### Luminescence assays.

For biofilm colonies, *B. subtilis* cultures were grown overnight and resuspended in LB-Lennox to an OD_600_ of 0.5, and then 10 μl of culture was spotted onto MSgg plates. Colonies were incubated at 30°C. Images were taken at 24 h using a ChemiDoc Touch imaging system (Bio-Rad, Hercules, CA) where the exposure time was set to 20 s, and the spectrum color map was applied to the images to detect intensity throughout the colonies. For liquid cultures, *B. subtilis* grown overnight at 30°C for 16 to 20 h was resuspended in LB-Lennox to an OD_600_ of 1.0, and then a 1:100 dilution into MSgg was performed and cultures were incubated with shaking at 28°C. Luminescence was measured using a SpectraMax L microplate reader (Molecular Devices, Sunnyvale, CA), and data were normalized by the absorbance at OD_600_. ImageJ 1.49v (49) was used to quantify luminescence. Statistical analysis was performed using two-tailed Student’s *t* tests.

### Flow cytometry.

*B. subtilis* cultures (10 μl) were spotted at an OD_600_ of 0.5 onto MSgg plates and incubated at 30°C for 24 h. Cells for flow cytometry were prepared by collecting the colony and suspending it in 1 ml 1× PBS and breaking up the colony using a needle and syringe. Cells were spun at 16,000 × *g* for 1 min, and the supernatant was removed. Cells were resuspended in 200 μl 4% (wt/vol) paraformaldehyde, incubated for 7 min at room temperature, and then spun at 16,000 × *g* for 1 min. Cells were washed with 1 ml 1× PBS, spun at 16,000 × *g* for 1 min, resuspended in 1 ml of GTE buffer (1% [wt/vol] glucose–5 mM EDTA–1× PBS, pH 7.4), and stored at 4°C. On the day of fluorescence quantification by flow cytometry, cells were sonicated for 12 pulses lasting 1 s each with 1-s pauses. Cells were filtered through a 38-μm-pore-size nylon mesh, and YFP fluorescence was measured using an LSR II flow cytometer (BD Biosciences). Statistical analysis was performed by one-way ANOVA with a Tukey test for multiple comparisons.

### Surfactin drop-collapse assay.

*B. subtilis* cells grown overnight (16 to 20 h) on LB agar plates at 30°C were resuspended in MSgg broth to an OD_600_ of 0.05 and then incubated in a roller at 37°C for 24 h. Cultures were spun down in a centrifuge, the supernatant was collected, and the cells were removed by the use of a 0.2-μm-pore-size filter. Crystal violet (0.01%) was added to the filtrate (cell-free spent media), 20 μl was spotted onto an empty petri dish and allowed to dry at room temperature, and then the diameter of the spread drop was measured. Statistical analysis was performed using two-tailed Student’s *t* tests.

### Plant root colonization.

The plant colonization experiments were performed as previously described (2) with slight modifications. *B. subtilis* was grown overnight (16 to 20 h) on LB agar plates at 30°C, cells were resuspended to an OD_600_ of 0.02 in MSNg (5 mM potassium phosphate buffer [pH 7], 0.1 M MOPS [pH 7], 2 mM MgCl_2_, 0.05 mM MnCl_2_, 1 μM ZnCl_2_, 2 μM thiamine, 700 μM CaCl_2_, 0.2% NH_4_Cl, 0.05% glycerol), and then 400 μl was added to each well of a 48-well plate (Becton, Dickinson Labware, Franklin Lakes, NJ). *A. thaliana* Col-0 seeds were surface sterilized and stratified for 4 days at 4°C as previously described by Vogel et al. (50). Six-day-old seedlings that had germinated on agar plates were placed into each well and allowed to incubate under conditions of 9 h of light at 21°C and 15 h of dark at 18°C. Plants were removed from wells, and roots were removed and gently washed with fresh MSNg and then placed on a microscope slide for imaging. Root attachment images were taken with a Zeiss-710 laser scanning microscope (LSM) (Zeiss, Oberkochen, Germany) or a Nikon Eclipse 80i epifluorescence microscope equipped with a Nikon Intensilight C-HGFI light source and with filters from Chroma Technology (Nikon, Tokyo, Japan) and were processed and linearly adjusted using ImageJ (49).
