# Supplementary figures and images for: Cyclic di-AMP Acts as an Extracellular Signal That Impacts Bacillus subtilis Biofilm Formation and Plant Attachment
Source: mBio. 2018 Mar 27;9(2):e00341-18. doi: 10.1128/mBio.00341-18 (PMC5874923; doi:10.1128/mBio.00341-18)

Townsley et al. Fig S3

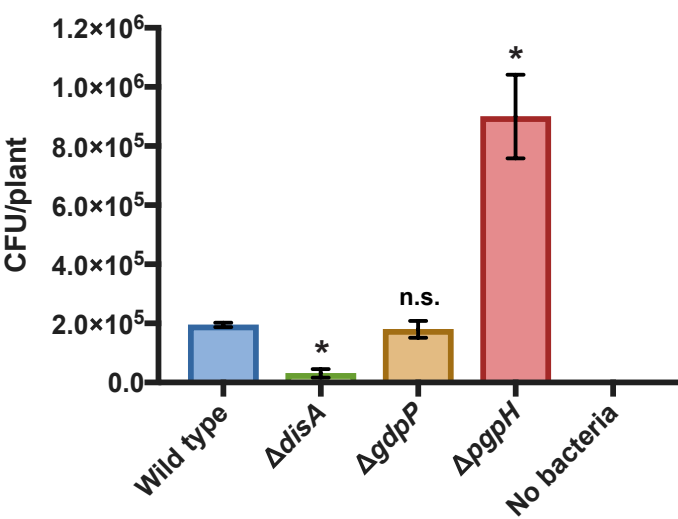

Supplement: FIG S3 [file mbo002183803sf3.pdf]

Townsley et al. Fig. S6

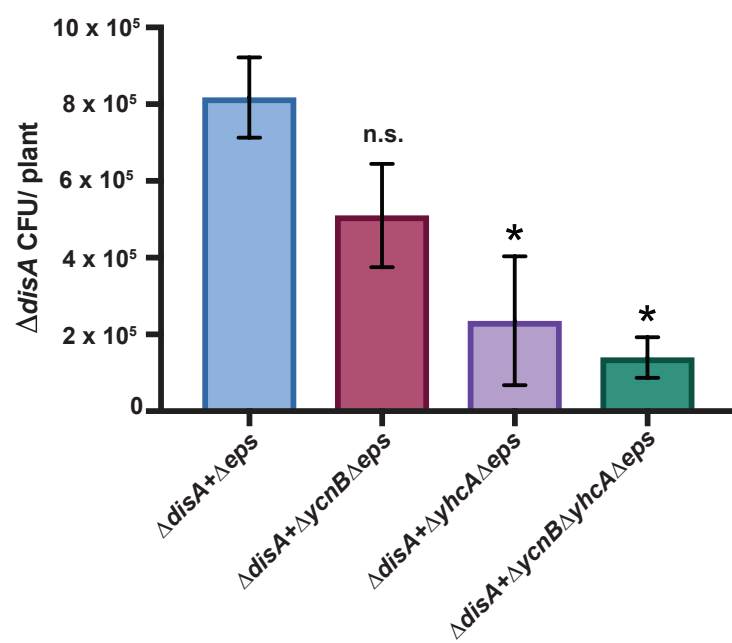

Supplement: FIG S6 [file mbo002183803sf6.pdf]
